# Serpine2/PN-1 Is Required for Proliferative Expansion of Pre-Neoplastic Lesions and Malignant Progression to Medulloblastoma

**DOI:** 10.1371/journal.pone.0124870

**Published:** 2015-04-22

**Authors:** Catherine Vaillant, Paola Valdivieso, Sandro Nuciforo, Marcel Kool, Alexandra Schwarzentruber-Schauerte, Hélène Méreau, Erik Cabuy, Johannes A. Lobrinus, Stefan Pfister, Aimée Zuniga, Stephan Frank, Rolf Zeller

**Affiliations:** 1 Developmental Genetics, Department of Biomedicine, University of Basel, Basel, Switzerland; 2 Division of Pediatric Neuro-Oncology, German Cancer Research Center DKFZ, Heidelberg, Germany; 3 Single Cell Genomics, Friedrich Miescher Institute, Basel, Switzerland; 4 Neuropathology Unit, Department of Clinical Pathology, University Hospital Geneva, Geneva, Switzerland; 5 Division of Neuropathology, Institute of Pathology, University of Basel, Basel, Switzerland; University Hospital of Navarra, SPAIN

## Abstract

**Background:**

Medulloblastomas are malignant childhood brain tumors that arise due to the aberrant activity of developmental pathways during postnatal cerebellar development and in adult humans. Transcriptome analysis has identified four major medulloblastoma subgroups. One of them, the Sonic hedgehog (SHH) subgroup, is caused by aberrant Hedgehog signal transduction due to mutations in the *Patched1* (*PTCH1*) receptor or downstream effectors. Mice carrying a *Patched-1* null allele (*Ptch1* ^∆/+^) are a good model to study the alterations underlying medulloblastoma development as a consequence of aberrant Hedgehog pathway activity.

**Results:**

Transcriptome analysis of human medulloblastomas shows that *SERPINE2*, also called *Protease Nexin-1* (*PN-1*) is overexpressed in most medulloblastomas, in particular in the SHH and WNT subgroups. As siRNA-mediated lowering of *SERPINE2/PN-1* in human medulloblastoma DAOY cells reduces cell proliferation, we analyzed its potential involvement in medulloblastoma development using the *Ptch1* ^∆/+^ mouse model. In *Ptch1* ^∆/+^ mice, medulloblastomas arise as a consequence of aberrant Hedgehog pathway activity. Genetic reduction of *Serpine2/Pn-1* interferes with medulloblastoma development in *Ptch1* ^∆/+^ mice, as ~60% of the pre-neoplastic lesions (PNLs) fail to develop into medulloblastomas and remain as small cerebellar nodules. In particular the transcription factor *Atoh1*, whose expression is essential for development of SHH subgroup medulloblastomas is lost. Comparative molecular analysis reveals the distinct nature of the PNLs in young *Ptch1* ^∆/+^ *Pn-1* ^Δ/+^ mice. The remaining wild-type *Ptch1* allele escapes transcriptional silencing in most cases and the aberrant Hedgehog pathway activity is normalized. Furthermore, cell proliferation and the expression of the cell-cycle regulators *Mycn* and *Cdk6* are significantly reduced in PNLs of *Ptch1* ^∆/+^ *Pn-1* ^Δ/+^ mice.

**Conclusions:**

Our analysis provides genetic evidence that aberrant *Serpine2/Pn-1* is required for proliferation of human and mouse medulloblastoma cells. In summary, our analysis shows that Serpine2/PN-1 boosts malignant progression of PNLs to medulloblastomas, in which the Hedgehog pathway is activated in a SHH ligand-independent manner.

## Introduction

Medulloblastomas are malignant cerebellar tumors belonging to the primitive neuroectodermal tumors that correspond to about one quarter of all primary brain tumors in children and young adults [1,2]. Deregulation of the Hedgehog (HH), NOTCH and WNT signaling pathways has been causally linked to the initiation of medulloblastomas in humans and mouse models [3]. In addition to these developmental regulators, amplification and/or over-expression of *MYCN* and *MYCC* are required for malignant progression. Genome-wide analyses showed that fewer genes are altered in childhood medulloblastomas than in adult solid tumors [4]. The most frequent inactivating mutations affect histone-lysine N-methyltransferases (*MLL2/MLL3*, 16%), which regulate developmental genes, the Sonic hedgehog (SHH) receptor *Patched-1* (*PTCH1*, 17%), and the WNT signal transducer *ß-catenin* (*CTNNB1*, 13%) [4–8]. Genome-wide analyses support the proposal that medulloblastomas arise as a consequence of deregulated cerebellar development [9] and allow molecular distinction of four medulloblastoma subgroups [6,10,11]. Most relevant to this study are the WNT and SHH subgroups, which arise from mutations in genes functioning in signal transduction and/or as nuclear effectors. In particular, human medulloblastomas of the SHH subgroup, accounting for ~30% of all medulloblastomas, arise mostly due to mutations in the *PTCH1* receptor, the transmembrane activator *Smoothened*, the signal transduction modulator *SUFU* and transcriptional regulator *GLI2* [6,7,12]. Mice heterozygous for a *Ptch1* null allele (*Ptch1* ^Δ/+^) provide a good animal model to study SHH subgroup medulloblastomas [13]. Normally, when the PTCH1 receptor is not complexed with the SHH ligand, it inhibits Smoothened (Smo) and thereby Hedgehog signal transduction [14] and mice expressing a constitutively active *Smo* transgene also rapidly develop medulloblastomas [15,16]. These medulloblastomas arise from granule neuron progenitors (GNP) [17] and express the *Atoh1* transcription factor, which is essential for development of medulloblastomas in mice as its inactivation suppresses medulloblastoma development [18–22]. During the first 3 weeks of postnatal cerebellar development in mice, the GNP population is expanded by proliferation and differentiation initiated within the external granular layer (EGL) [23]. Then, the differentiating granule neurons migrate through the molecular layer (ML) to their destinations in the internal granular layer (IGL) and *Atoh1* expression is terminated [24]. In *Ptch1* ^Δ/+^ mice, proliferative lesions are detected in the outer EGL already at postnatal day 10, which will give rise to pre-neoplastic lesions (PNLs) [17]. Pre-neoplastic cells continue to express *Atoh1* and the remaining wild-type *Ptch1* allele is transcriptionally silenced by DNA methylation [19,25]. This transcriptional silencing of *Ptch1* is a pre-requisite for malignant progression of PNLs to medulloblastomas in *Ptch1* ^Δ/+^ mice [19,25,26]. Another important alteration involves *Mycn*, a transcriptional target of HH signaling, whose stable over-expression is key to malignant progression [25–28]. Overexpression of *Mycn* in PNL cells renders medulloblastomas resistant to HH pathway antagonists [26], while its inhibition induces senescence [28].

The extra-cellular Serine protease inhibitor E2 (Serpine2), also called Protease Nexin-1 (PN-1) belongs to the *Serpin* gene superfamily. *Serpins* are expressed in tissues throughout the body and function in many physiological processes including inflammation, tumor growth and metastasis [29,30]. In particular, Serpine2/PN-1 is up-regulated in a large number of invasive/metastatic tumors including breast, prostate, pancreatic, colorectal, oral-squamous, and testicular cancers and is required for tumor growth and malignant progression [31–35]. Serpine2/PN-1 is up-regulated by ERK signal transduction and forms covalent complexes with its protease substrates in the extra-cellular matrix (ECM) following secretion [32]. These complexes interact with the LRP1 receptor, which enhances ERK signal transduction and expression of the matrix-metalloprotease MMP9 [33]. In turn, MMP9 cleaves Serpine2/PN-1, which enables protease-mediated remodeling of the ECM [36]. These complex feedback interactions not only promote tumor growth, but also invasion and metastasis of tumor cells in mouse xenograft models [37]. In contrast, the analysis of *Serpine2/Pn-1*-deficient mice revealed that during normal cerebellar development, Serpine2/PN-1 interacts with the low-density lipoprotein receptor related protein-1 (LRP-1) to inhibit GNP proliferation controlled by SHH ligands [38]. Likewise, LRP1-mediated internalization of SERPINE2/PN-1 inhibits SHH ligand-dependent signaling in human prostate adenocarcinoma cells, which results in down-regulation of *SHH* and *GLI1* expression [36]. This reduces proliferation and interferes with SHH-dependent prostate tumor growth in a mouse xenograft model. These authors show that MMP-9 promotes SHH signaling and tumor growth indirectly by cleaving SERPINE2/PN-1 and propose that increasing its levels may be critical for blocking malignant progression of prostate cancer [36]. Taken together, these studies reveal the complexity of Serpine2/PN-1 functions during normal development and tumorigenesis.

Our initial comparative analysis of the transcriptomes of human medulloblastomas and other brain tumors showed that *SERPINE2/PN-1* is expressed at high levels in the WNT and SHH subgroups. siRNA-mediated downregulation of SERPINE2/PN-1 in human DAOY medulloblastoma cells reduced their proliferation. To gain further insight, we used the *Ptch1* ^Δ/+^ mouse model and first established that Serpine2/PN-1 is overexpressed in PNLs and medulloblastomas. Heterozygosity for *Serpine2/Pn-1* (*Pn-1* ^Δ/+^) reduces the frequency of medulloblastomas in *Ptch1* ^Δ/+^ mice by ~60%. Histological and molecular analysis of the large number of surviving *Ptch1* ^Δ/+^ *Pn-1* ^Δ/+^ mice at 10–11 months shows that only ~20% of them display small cerebellar nodules. These lack *Atoh1* expression and MMP9 is reduced in comparison to *Ptch1* ^Δ/+^ medulloblastomas, while the expression of some differentiation markers is increased. Molecular analysis of PNLs in both genotypes at 6 weeks revealed that the remaining wild-type *Ptch1* allele is not silenced in the majority of *Ptch1* ^Δ/+^ *Pn-1* ^Δ/+^ mice, which is consistent with the significant reduction in aberrant Hedgehog signal transduction. In addition, the expression of cell cycle regulators and cell proliferation itself are reduced by ~50% in *Ptch1* ^Δ/+^ *Pn-1* ^Δ/+^ PNLs compared to their *Ptch1* ^Δ/+^ counterparts. This genetic analysis in mice shows that Serpine2/PN-1 is required for proliferation of PNL cells and malignant progression to medulloblastomas in the context of SHH ligand-independent up-regulation of Hedgehog pathway activity.

## Materials and Methods

### Ethics Statement

The histological analysis of all human medulloblastoma biopsies was authorized by the ethics committees on human studies of the cantons of Basel and Geneva. The ethics committees waived the need for consent, but the guidelines of the ethics committees of Basel and Geneva (www.eknz.ch; www.ethiquerecherche.hug-ge.ch) were strictly followed for the analysis of all human samples. All studies involving mice were performed in strict accordance with Swiss law after approval by the Joint Commission on Experiments involving Animals of Argovia and both Cantons of Basel (Gemeinsame Tierversuchskommission der Kantone Aargau, Basel-Land und Basel-Stadt). The relevant license no. 2265 entitled ‘‘Modulation of SHH pathway activity: potential effects of medulloblastoma formation and progression” was issued by the Veterinary Office of Basel (valid until 01.01.2017). The 3R principles were implemented as mandated by Swiss law.

### Metadata Analysis of Human Medulloblastoma Microarrays

The gene expression analysis shown for candidate genes in medulloblastoma, other tumours and normal tissues was compiled from multiple gene expression profiling studies [5,39–47] (Kool et al. unpublished data). All samples were analysed using the Affymetrix GeneChip Human Genome U133 Plus 2.0 arrays. The MAS5.0 algorithm of the GCOS program (Affymetrix Inc.) was used to normalize the expression data. Data were analysed and statistically verified using the R2 software platform for analysis and graphic visualization of microarray data (see http://r2.amc.nl).

### Immunohistochemistry on Human Medulloblastoma Biopsies

Human medulloblastoma biopsies (n = 43) were collected and archived as formalin-fixed paraffin blocks by the Institute of Pathology at the University of Basel and the Department of Clinical Pathology at the University of Geneva between 1989 and 2010. For details of ethical approval see the ethics statement before. All except two samples were identified as classic medulloblastomas according to the WHO classification of brain tumors. Histological and immunohistochemical analysis was performed as described below for mouse samples.

### siRNA-mediated Reduction of SERPINE2/PN-1 in DAOY Cells

The human medulloblastoma cell line DAOY (ATCC no. HTB-186) was maintained in complete growth medium (Opti-MEM, 10% FBS, 100 U/ml Penicillin, 100 μg/ml Streptomycin, Invitrogen) at 37°C in 5% CO_2_ and passaged at 90% confluency. Cells were plated 24 hours before transfection into 6-well plates at a density of 10^5^ cells/well in 2 ml culture medium. siRNA oligos against human PN-1 were designed against previously experimentally validated target sequences resulting in effective reduction of endogenous PN-1 [33]: human siPN-1: 5’GCA GUG UGC CUG UCA CUA CUU3’. The scrambled oligo control corresponds to the following sequence: 5'FITC–GCC UCU UCG CCG AGA CAC UU3’. The pre-annealed siRNA oligos (Dharmacon RNAi Technologies) were transfected using mixture of 100pmol siRNA and 5μl Lipofectamine RNAiMAX (Invitrogen) per well (final siRNA concentration: 50 nM; transfection efficiency ~80%).

### Cellular Extracts for Immunoblotting

Cells were lysed in NP-40 lysis buffer (150mM NaCl, 1% NP-40, 50mM Tris-HCl, pH8.0, 0.4mM Pefabloc; 1x Complete Mini Protease Inhibitor Cocktail from Roche Diagnostics, 1 mM Na_3_VO_4_, 1mM NaF). The lysate was centrifuged for 10 minutes at 4°C and the protein concentration determined using the BCA Protein Assay Kit (Pierce). Ten μg total protein was separated by 10% SDS-PAGE and transferred to a PVDF membrane. Protein was detected using standard immunoblotting.

### Immunofluorescence

Cells on coverslips were fixed in 4% PFA for 10 minutes and then extensively washed with PBS. Histological sections of tissues fixed overnight in 4% PFA and embedded were dewaxed and rehydrated into PBS following standard procedures. After an initial incubation in 1% BSA; 0.3% Triton X-100 in PBS for 1 hour, antigens were detected by incubation with primary antibodies in blocking buffer for 2 hours and incubated secondary antibodies coupled to the appropriate fluorochromes (S2 Table) for 1.5 hours (all at room temperature). Cell nuclei were counterstained with DAPI or Hoechst 33258, slides mounted in Mowiol and analyzed using a confocal microscope. For all results shown, at least three independent samples per genotype were analyzed and results were identical.

### Cell Proliferation Analysis

DAOY cells on coverslips were pulsed for 1 hour with BrdU (final concentration: 10 μM) and fixed in 4% PFA for 10 minutes. Then cells were incubated in 1N HCl for 10 minutes, 2N HCl for 10 minutes (all at room temperature), and finally at 37°C for 20 minutes. This was followed by incubation in 0.1M borate buffer (pH 8.5) for 12 min and monoclonal G3G4 anti-BrdU antibodies (dilution of 1:1000, S2 Table) for 2 hours at room temperature. Immunofluorescent detection was done as described above. Mean and standard deviations of the data points for each sample were determined and graphs drawn using the GraphPad Prism software. This software was used to calculate p-values by the Student’s test.

### Mice

The murine *Ptch1* ^Δ/+^ constitutive loss-of-function (null allele) [13] was imported from Jackson labs and a breeding colony in the C57BL6/J background established. To generate *Ptch1* ^Δ/+^ *Pn-1* ^Δ/+^ mice, *Ptch1* ^Δ/+^ males were crossed with *Pn-1* ^Δ/Δ^ females [38] in the C57BL6/J background. To generate *Ptch1* ^Δ/+^ *Pn-1* ^Δ/Δ^ mice, *Ptch1* ^Δ/+^ *Pn-1* ^Δ/+^ mice were crossed again with *Pn-1* ^Δ/Δ^ females. No causes of lethality other than medulloblastomas were observed in *Ptch1* ^Δ/+^ *Pn-1* ^Δ/+^ and *Ptch1* ^Δ/+^ *Pn-1* ^Δ/Δ^ mice. All mice carrying a *Ptch1* null-allele were monitored daily for their well-being and symptoms of medulloblastoma development over 11 months. Mice displaying signs of discomfort and/or motor dysfunction were euthanized immediately, autopsied and medulloblastomas analyzed.

### Histology, Immunohistochemistry and RNA *in situ* Hybridization

Adult mouse brains were fixed in fresh 4% paraformaldehyde at 4°C overnight, washed, dehydrated, and embedded in paraffin. Eight μm paraffin sections were mounted on Superfrost slides (Menzel Glas, Germany), air-dried and stored at 4°C. Following dewaxing and rehydration, sections were either stained with Cresyl-violet or treated with 10mM citrate buffer (pH 6.0) in a pressure cooker for 6 minutes to recover antigenicity. Endogenous peroxidases were inactivated in 0.3% H_2_O_2_ in methanol for 30 minutes (room temperature). Non-specific binding was blocked by saturating sections with blocking buffer for one hour (PBS, 0.3% Triton X-100, 1% BSA at room temperature). Sections were incubated with primary antibodies in blocking buffer overnight (4°C) and with biotinylated secondary antibodies for 2 hours (room temperature; S2 Table). Immune-complexes were detected using the Elite ABC and Impact DAB substrate kits (Vector Laboratories). Sections were counterstained with Hoechst 33258 (5mg/ml) for 1 minute and mounted with Mowiol (Calbiochem). RNA *in situ* hybridization analysis was done as described previously [38]. For all results shown, at least three independent samples per genotype were analyzed.

### Laser Dissection of PNL Tissue from Frozen Sections for qPCR Analysis

Adult mouse brains were immersed in Tissue Tek-OCT (Sakura), flash-frozen in isopentane and 20μm serial cryostat sections prepared. Three consecutive sections with PNL tissue were collected on a Membrane Slide (MMI) and counterstained briefly with Haematoxylin (MMI) prior to dehydration in 100% ethanol and air-drying. The PNL tissue was laser-dissected using a MMI CellCut-Plus laser capture microscope. Tissue corresponding to ~120’000μm^2^ from each of the 3 sections was pooled into one MMI Isolation Cap tube, homogenized for 15 min in 50μl of PicoPure RNA extraction buffer (Arcturus, room temperature) and the lysate stored at -20°C. Total RNA was extracted using the PicoPure RNA isolation kit (Arcturus) and cDNA synthesized using the SuperScript-III kit (Invitrogen). qPCR analysis was done as described previously and transcript levels were normalized in comparison to *Hprt1*, a housekeeping gene (for more details see ref. [48]). For qPCR primers see S3 Table. The mean, standard deviations and p-values (Student’s t-test) for all samples were determined and the graphs drawn using the GraphPad Prism software.

## Results

### SERPINE2/PN-1 is over-expressed in human medulloblastoma biopsies and the *Ptch1* ^Δ/+^ mouse model

**Fig 1:**
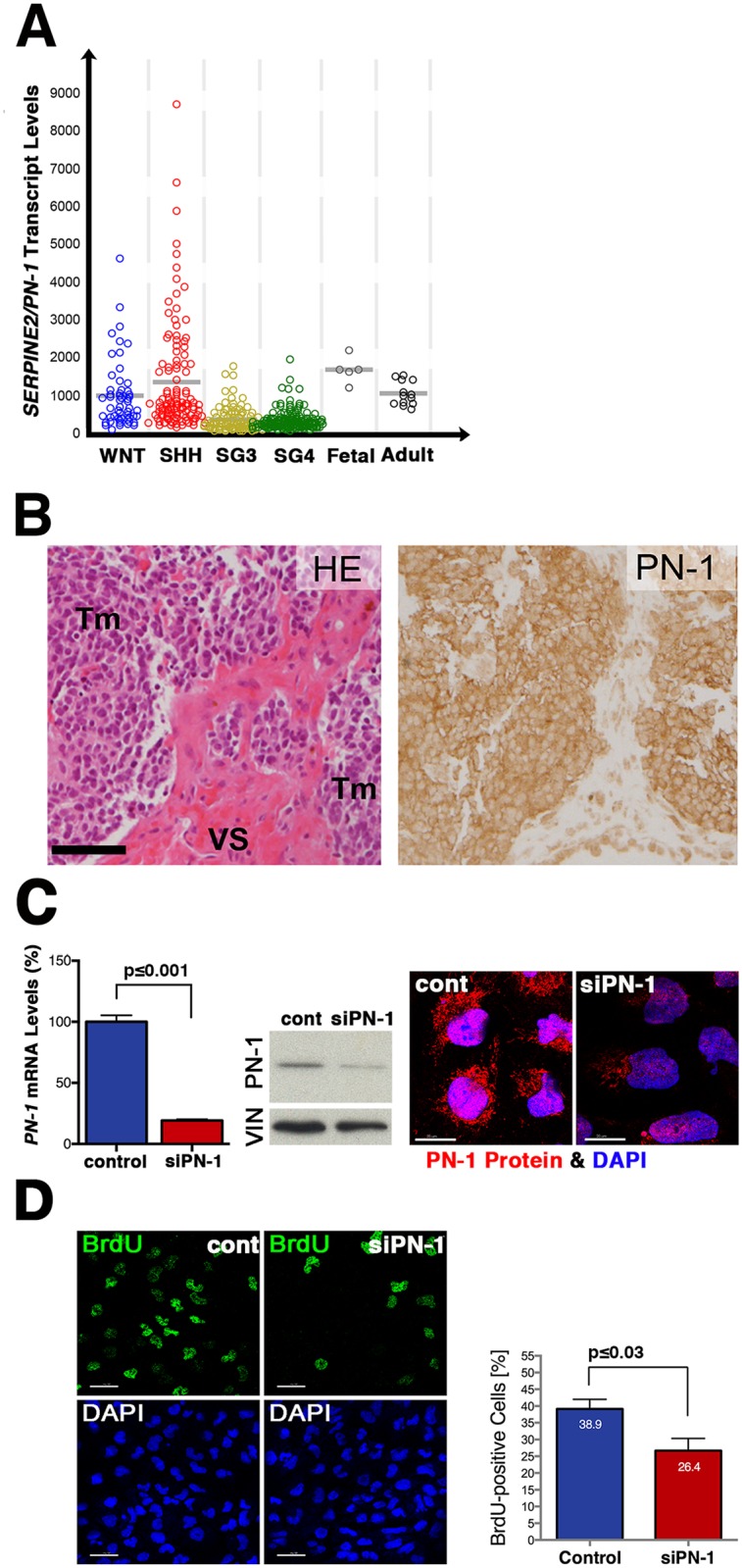
Overexpression of SERPINE2/PN-1 in human medulloblastomas. (A) SERPINE2/PN-1 transcript levels in human medulloblastoma subgroups in comparison to normal fetal and adult cerebellum. Data for medulloblastoma (n = 437) and normal controls (n = 18) were generated by Affymetrix Human U133 Plus2.0 arrays and were MAS5.0 normalized. Grey bars represent mean values. (B) Analysis of serial sections of a representative human medulloblastoma biopsy. Left Panel: haematoxylin-eosin (HE) staining reveals the high cellularity of the medulloblastoma biopsy. Right Panel: high levels of cytoplasmic SERPINE2/PN-1 protein are detected in tumor cells. Tm: tumor tissue, VS: vascular stromal cells. Scale bar: 150μm. (C) In DAOY cells, SERPINE2/PN-1 is downregulated by transfection of a verified PN-1 siRNA. 72 hours following transfection, SERPINE2/PN-1 transcript levels are reduced by ≥80% (n = 3; p≤0.001) and protein levels by ≥50% (upper left and middle panels, n = 3). Controls are DAOY cells treated with scrambled siRNA, but treated otherwise identical to experimental samples. VIN: vinculin was used to normalize immunoblots for protein content. Right panels: immunofluorescence analysis reveals the reduction of SERPINE2/PN-1 proteins (red fluorescence). Nuclei appear blue due to counterstaining with DAPI. (D) siPN-1 transfection results in reduced BrdU incorporation into DAOY cells at 72 hours (1-hour pulse). The left panels show representative fields of the fraction of BrdU-positive nuclei (green, upper panels) in siPN-1 and control cells (lower panels reveal all nuclei). Quantification revealed that the number of BrdU-positive cells is decreased by ≥30% in DAOY cells (n = 3, p≤0.03). Scale bars: 20μm.

Molecular analysis has identified at least four distinct subgroups of human medulloblastomas called the SHH, WNT and Group 3 and 4. In particular, the SHH and WNT subgroups are characterized by aberrant activity of the WNT and Hedgehog signaling pathways. Metadata analysis of microarray data from 437 human medulloblastoma samples (see Materials and Methods) establishes that *SERPINE2/PN-1* is expressed by the vast majority of all medulloblastomas, with levels highest in the WNT and SHH subgroups (Fig 1A). In addition, *SERPINE2* is also expressed by a variety of other pediatric and adult brain tumors, most prominently in glioblastomas and different forms of astrocytomas (S1 Fig, refs. [5,39–47] and Kool et al. unpublished data). In contrast, *SERPINE1* is expressed at much lower levels and no differences are observed between medulloblastoma subgroups (data not shown).

In agreement with the transcriptome analysis, SERPINE2/PN-1 protein is expressed abundantly in medulloblastoma biopsies (Fig 1B, see also S2 Fig). Metadata analysis of the SHH medulloblastoma subtype revealed 410 genes whose expression correlates significantly with PN-1 (S1 Table). In addition to the SHH receptor *PTCH1* and the transcriptional regulator *SOX9* (S1 Fig, see also below), the expression of cell-cycle regulators positively correlated with SERPINE2/PN-1 in medulloblastomas (S1 Table and data not shown). To gain first insights into possible SERPINE2/PN-1 functions in medulloblastoma cells, we used a siRNA-based approach to lower its expression in human DAOY cells (Fig 1C and 1D), which are a cellular model for SHH subgroup medulloblastomas [49]. In particular, the HH pathway is active in DAOY cells (data not shown) and inhibition of HH signal transduction lowers their tumorigenic potential [50]. Transfection of DAOY cells with a previously verified *SERPINE2/PN-1* specific siRNA (siPN-1) [33] reduces transcripts by about ~80% from 24 hours onward and protein levels by ≥50% at 72 hours (Fig 1C, n = 3). This siRNA-mediated reduction of SERPINE2/PN-1 is paralleled by a significant decrease of BrdU incorporation into DAOY cells at 72 hours (Fig 1D). Quantitation of BrdU-positive cells shows that treatment of DAOY cells with *PN-1* siRNA decreases their proliferation ≥30% in comparison to control cells transfected with a scrambled siRNA (Fig 1D, n = 3, p<0.01).

**Fig 2:**
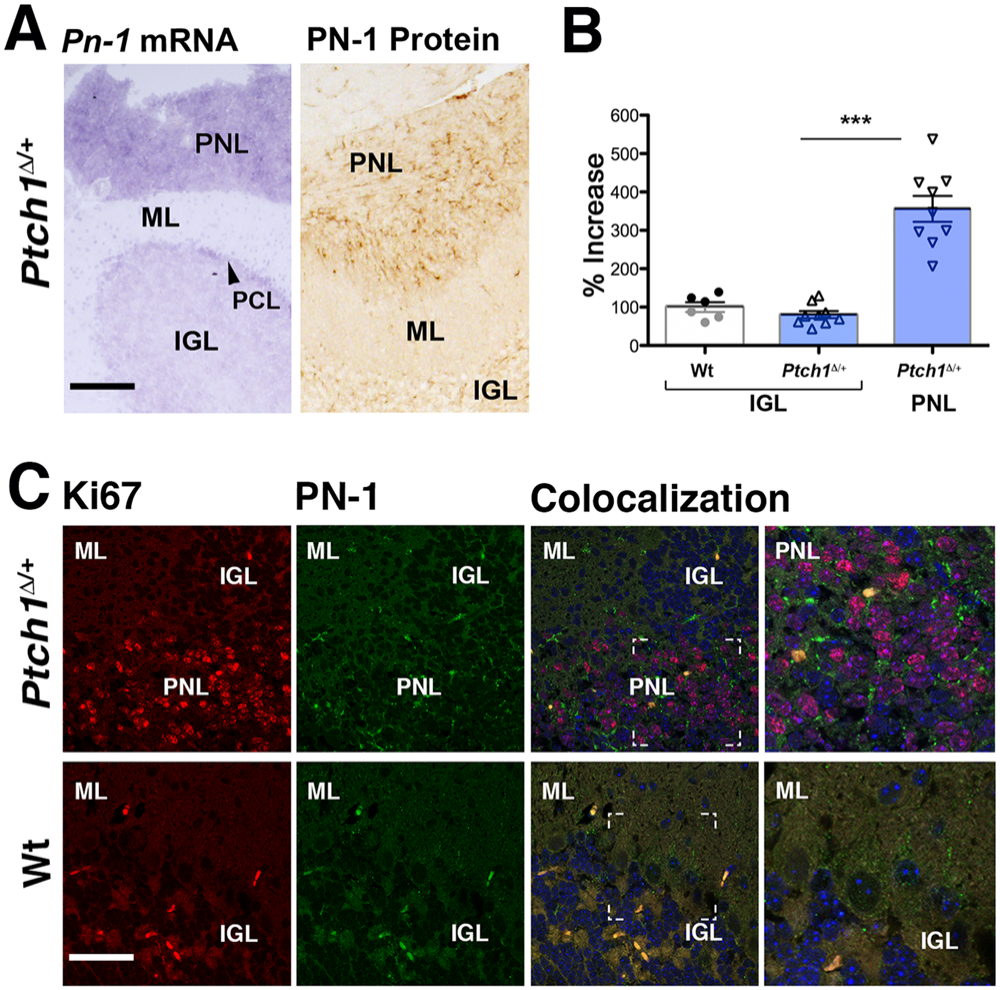
Serpine2/PN-1 is overexpressed in cerebellar PNLs of *Ptch1* ^Δ/+^ mice. (A) Left panel: *Serpine2/Pn-1* transcript distribution (purple staining of the RNA hybrids) in the cerebellum of a *Ptch1* ^Δ/+^ mouse 6 weeks after birth. *E*xpression is highest in the pre-neoplastic lesions (PNL) within the cerebellum. Right panel: Serpine2/PN-1 protein levels (brown staining of the immunocomplexes) are highest in the PNLs of *Ptch1* ^Δ/+^ mice. (B) qPCR analysis of *Serpine2/Pn-1* transcripts in laser-dissected tissue from wild-type (Wt) and *Ptch1* ^Δ/+^ normal IGL tissues in comparison to *Ptch1* ^Δ/+^ PNLs. Expression in the wild-type IGL was set to 100% (S3 Table), p≤0.001. (C) Co-localization of Ki67-positive proliferating cells (red fluorescence) and Serpine2/PN-1 (green fluorescence) in the cerebellum of wild-type and *Ptch1* ^Δ/+^ mice at 6 weeks (n = 3). White lines indicate the position of the enlargement shown in the right-most panel. Auto-fluorescent cells are detected equally in the red and green channel and appear yellow-orange in the overlap (Colocalization, right panels). IGL: internal granular layer; ML: molecular layer; PCL: Purkinje cell layer; PNL: pre-neoplastic lesion. Scale bars: 100μm.

To gain further insight into the potential involvement of Serpine2/PN-1 in medulloblastoma development, we took advantage of *Ptch1* heterozygous (*Ptch1* ^Δ/+^) mice [51]. We assessed the Serpine2/PN-1 transcript and protein expression during these early stages of medulloblastoma development (Fig 2 and S3 Fig). Indeed, *Serpine2/Pn-1* transcript levels were increased in PNLs of *Ptch1* ^Δ/+^ mice from postnatal week 3 onward (Fig 2A, 2B and S3 Fig). Furthermore, Serpine2/PN-1 protein was over-expressed in all PNLs of *Ptch1* ^Δ/+^ mice as revealed by immunohistochemistry 6 weeks postnatally (Fig 2A and S3 Fig). *Serpine2/Pn-1* transcript levels were quantified by real-time qPCR analysis of laser-dissected tissues. In contrast to the wild-type and *Ptch1* ^Δ/+^ internal granular layer (IGL), *Serpine2/Pn-1* transcript levels are ~3.4 fold higher in PNLs of *Ptch1* ^Δ/+^ mice (Fig 2B). Simultaneous detection of Ki67-positive cells (red nuclei) and Serpine2/PN-1 protein (green dots) indicates that regions of proliferating PNL cells overlap with Serpine2/PN-1 expression domains in the cerebellum of *Ptch1* ^Δ/+^ mice (Fig 2C). At 6 weeks of age, only low levels of Serpine2/PN-1 and few to no Ki67-positive cells are detected in wild-type cerebella (lower panels, Fig 2C), which underscores the aberrant Serpine2/PN-1 expression by proliferating PNL cells in *Ptch1* ^Δ/+^ mice (upper panels, Fig 2C).

**Fig 3:**
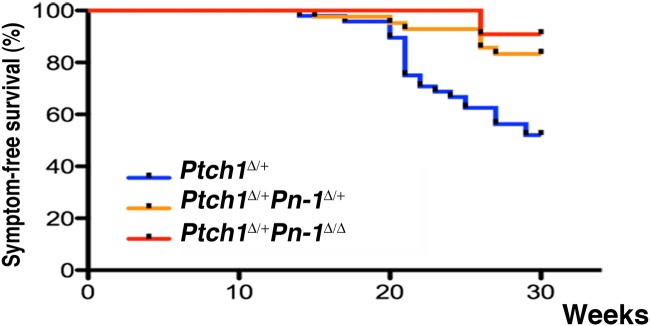
Genetic reduction of *Serpine2/Pn-1* (*Pn-1* ^Δ^ allele) increases the long-term symptom-free survival of *Ptch1* ^Δ/+^ mice. Kaplan-Meier curves establish that genetic inactivation of one or both *Pn-1* alleles reduces the development of end-stage medulloblastomas in *Ptch1* ^Δ/+^ mice, significantly improving their long-term symptom-free survival (log-rank Mantel Cox test, p = 0.0016).

This potential role of Serpine2/PN-1 during medulloblastoma development in *Ptch1* ^Δ/+^ mice was assessed genetically by inactivating one or both copies of the *Serpine2/Pn-1* gene using a constitutive loss-of-function (*Pn-1* ^Δ^) allele [38]. Littermates from all three genotypes were monitored daily for symptoms and the presence of medulloblastomas was confirmed by autopsy (Fig 3). During the monitoring period, more than one third of *Ptch1* ^Δ/+^ mice developed medulloblastomas by week 30 (n = 23/58, 39%), while this frequency was reduced by ≥60% in *Ptch1* ^Δ/+^ *Pn-1* ^Δ/+^ (n = 7/42, 16%) and *Ptch1* ^Δ/+^ *Pn-1* ^Δ/Δ^ mice (n = 1/11, 9%). This reduction improved the overall symptom- and tumor-free survival of compound mutant mice. As the frequency of medulloblastomas was not decreased much more in *Ptch1* ^Δ/+^ *Pn-1* ^Δ/Δ^ than *Ptch1* ^Δ/+^ *Pn-1* ^Δ/+^ mice (Fig 3, compare orange to red line), the subsequent analysis focused on *Ptch1* ^Δ/+^ *Pn-1* ^Δ/+^ mice.

### Medulloblastoma development is reduced in *Ptch1* ^Δ/+^ *Pn-1* ^Δ/+^ mice

**Fig 4:**
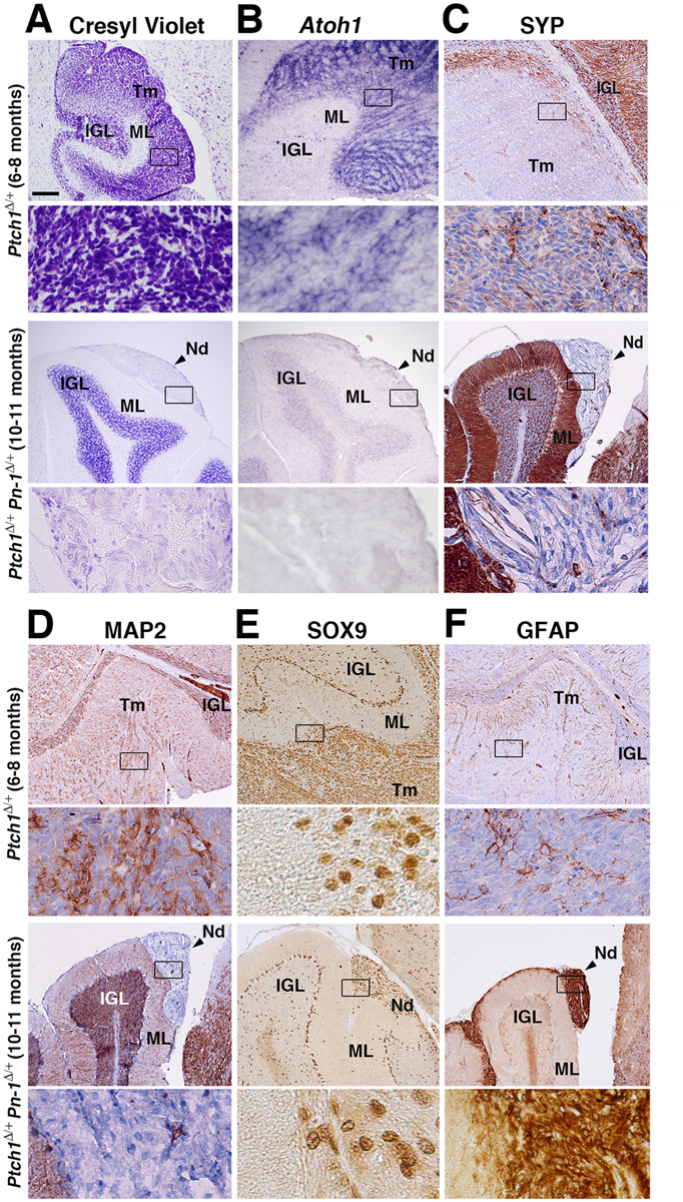
Comparative analysis of end-stage medulloblastomas and cerebellar nodules in *Ptch1* ^Δ/+^ and *Ptch1* ^Δ/+^ *Pn-1* ^Δ/+^ mice. (A-F) Representative analysis of end-stage *Ptch1* ^Δ/+^ medulloblastomas at 6–8 months (upper panels) and the superficial cerebellar nodules (Nd) detected in a fraction of *Ptch1* ^Δ/+^ *Pn-1* ^Δ/+^ mice at 10–11 months of age (lower panels). (A) Cresyl violet staining to reveal cerebellar morphology. Upper panels: A large number of cells are present in the molecular layer (ML) of the *Ptch1* ^Δ/+^ cerebellum, which is indicative of the massive growth and invasion of the end-stage medulloblastoma. In contrast, the cerebellar nodules of *Ptch1* ^Δ/+^ *Pn-1* ^Δ/+^ mice remain superficially restricted, i.e. do not invade the cerebellar cortex. This is confirmed by molecular analysis (panels B-F and S4 Fig). (B) Distribution of *Atoh1* transcripts revealed by RNA *in situ* hybridization (purple). Note the abundant expression in *Ptch1* ^Δ/+^ medulloblastomas whereas it is not detected in the cerebellar nodules of *Ptch1* ^Δ/+^ *Pn-1* ^Δ/+^ mice. (C, D) Synaptophysin (SYP) and MAP2 proteins are both expressed in *Ptch1* ^Δ/+^medulloblastomas, but are absent from cerebellar nodules in *Ptch1* ^Δ/+^ *Pn-1* ^Δ/+^ mice. (E) Distribution of the SOX9 transcriptional regulator. The fraction of SOX9-positive cells varies among different *Ptch1* ^Δ/+^ medulloblastomas, while the expression is rather uniform in cerebellar nodules of *Ptch1* ^Δ/+^ *Pn-1* ^Δ/+^ mice. Bergmann glia and scattered cells in the IGL also express SOX9. (F) GFAP is expressed at low levels in *Ptch1* ^Δ/+^ medulloblastomas, while it is abundant in cerebellar nodules of *Ptch1* ^Δ/+^ *Pn-1* ^Δ/+^ mice (same nodule as shown in the lower panels of C, D). Protein immunocomplexes appear brown in all panels. Frames indicate the areas magnified in the panels below. IGL: internal granular layer; ML: molecular layer; Nd: cerebellar nodule; Tm: tumor tissue. Scale bar: 100μm (representative for all low magnifications shown).

**Fig 5:**
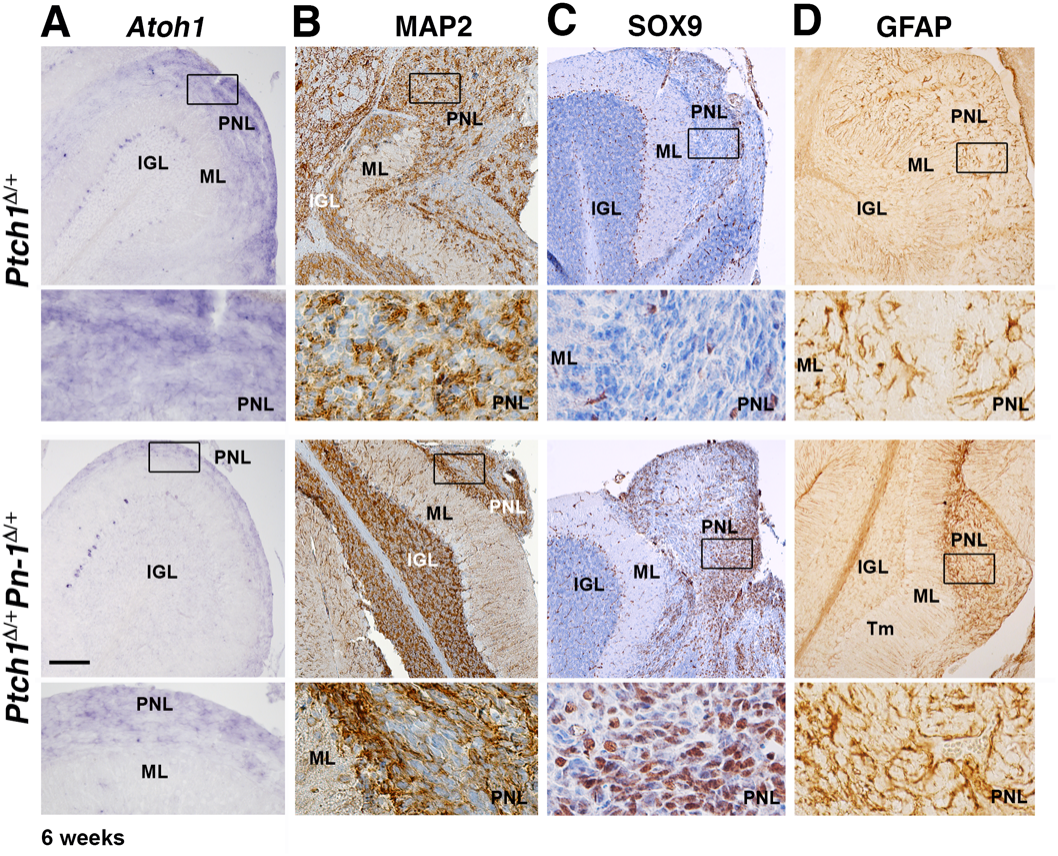
Comparative analysis of PNLs in both genotypes at 6 weeks of age. (A) *Atoh1* transcripts (purple) are detected in PNLs of *Ptch1* ^Δ/+^ and *Ptch1* ^Δ/+^ *Pn-1* ^Δ/+^ mice, which corroborates their GNP origin in both genotypes. (B) MAP2 is abundant in the PNL and normal IGL of both genotypes. (C) SOX9 protein. Only scattered SOX9-positive cells are detected in *Ptch1* ^Δ/+^ PNLs, while large areas of SOX9- positive cells are detected in *Ptch1* ^Δ/+^ *Pn-1* ^Δ/+^ PNLs. (D) GFAP expression is increased in PNLs of *Ptch1* ^Δ/+^ *Pn-1* ^Δ/+^ mice in comparison to adjacent normal tissue (IGL and ML). Protein immunocomplexes appear brown in all panels. Frames indicate the enlargements shown in the panels below. IGL: internal granular layer; ML: molecular layer; PNL: pre-neoplastic lesion. Scale bar: 100μm (representative for all low magnifications shown).

The cerebella of all mice surviving for 10–11 months were analyzed by serial sectioning, which confirmed the previously established lack of neoplastic lesions in surviving *Ptch1* ^Δ/+^ mice [13]. In contrast, small nodules located mostly in superficial positions in either lobe V or IX of the cerebellum were detected in ~20% of the surviving *Ptch1* ^Δ/+^ *Pn-1* ^Δ/+^ mice at 10–11 months (panels *Ptch1* ^Δ/+^ *Pn-1* ^Δ/+^, Fig 4, n = 5/24). In contrast to the end-stage medulloblastomas in *Ptch1* ^Δ/+^ mice at 6–8 months, these cerebellar nodules are much smaller and consist of larger, less densely packed cells (Fig 4A). Medulloblastomas in *Ptch1* ^Δ/+^ mice originate mostly from cells committed to the GNP lineage and all cells continue to express high levels of *Atoh1* transcripts, in agreement with their immature GNP origin and highly proliferative nature (Fig 4B) [22,52]. In contrast, *Atoh1* is no longer expressed by the cerebellar nodules in *Ptch1* ^Δ/+^ *Pn-1* ^Δ/+^ mice (Fig 4B). Therefore, we also assayed the expression of the GABA receptor alpha6-subunit (GABARα6), which marks mature cerebellar granule neurons (S4 Fig) [53]. However, no GABARα6-positive cells are detected in cerebellar nodules of *Ptch1* ^Δ/+^ *Pn-1* ^Δ/+^ mice, which renders their differentiation into granule neurons unlikely (S4 Fig). The synaptic vesicle protein Synaptophysin (SYP) and the microtubule-associated protein MAP2 are both expressed in *Ptch1* ^Δ/+^ medulloblastomas [20,21], but not in the cerebellar nodules of aged *Ptch1* ^Δ/+^ *Pn-1* ^Δ/+^ mice (Fig 4C and 4D). The fraction of SOX9-positive cells varies among *Ptch1* ^Δ/+^ medulloblastomas, likely due to their cellular heterogeneity (upper panels, Fig 4E, see also Fig 5C). In contrast, most cells of the cerebellar nodules in *Ptch1* ^Δ/+^ *Pn-1* ^Δ/+^ mice are SOX9-positive (lower panels, Fig 4F). High levels of the intermediate filament protein GFAP, which marks both normal and neoplastic glial cells, are detected in all *Ptch1* ^Δ/+^ *Pn-1* ^Δ/+^ nodules, while only low or no expression is detected in *Ptch1* ^Δ/+^ medulloblastomas (Fig 4F). Taken together, this molecular analysis points to the rather benign nature of the cerebellar nodules detected in ~20% of aged *Ptch1* ^Δ/+^ *Pn-1* ^Δ/+^ mice. This observation is in agreement with the long-term symptom-free survival of the vast majority of *Ptch1* ^Δ/+^ *Pn-1* ^Δ/+^ mice (Fig 3). All cerebellar nodules in *Ptch1* ^Δ/+^ *Pn-1* ^Δ/+^ mice remain superficially in the position of the former EGL, i.e. fail to grow and invade into the molecular layer and IGL as the case for end-stage medulloblastomas in *Ptch1* ^Δ/+^ mice (Fig 4 and S4 Fig). Taken together, ~36% of all *Ptch1* ^Δ/+^ *Pn-1* ^Δ/+^ mice develop either medulloblastomas (~16%) or small cerebellar nodules (~20%), which together correlates well with the frequency of medulloblastomas in *Ptch1* ^Δ/+^ mice (~39%). These observations indicate that the initiation of PNLs is similar in both genotypes, but that the malignant progression of PNLs to medulloblastomas is reduced by ~60% in *Ptch1* ^Δ/+^ *Pn-1* ^Δ/+^ mice. This reduction might be a consequence of cells losing *Atoh1* expression (Fig 4B) [22], which is paralleled by up-regulation of several differentiation markers (Fig 4C to 4F; S4 Fig).

**Fig 6:**
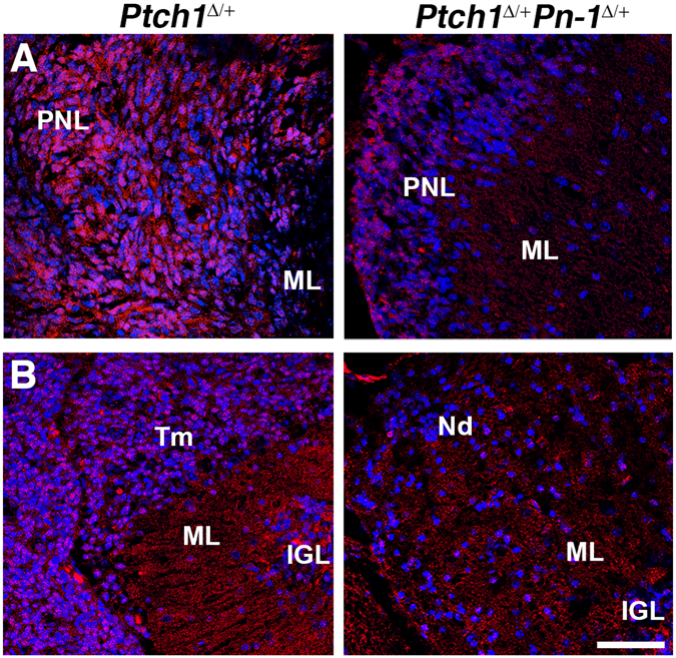
Distribution of the matrix-metalloprotease MMP9 in PNLs, *Ptch1* ^Δ/+^ medulloblastomas and *Ptch1* ^Δ/+^ *Pn-1* ^Δ/+^ cerebellar nodules. (A) Analysis of the extra-cellular MMP9 proteins (red fluorescent dots) in cerebella of *Ptch1* ^Δ/+^ and *Ptch1* ^Δ/+^ *Pn-1* ^Δ/+^ mice reveals its expression in PNLs. (B) MMP9 expression persists in *Ptch1* ^Δ/+^ medulloblastomas (left panel), while it is much lower in cerebellar nodules of *Ptch1* ^Δ/+^ *Pn-1* ^Δ/+^ mice (right panel). Nuclei appear blue due to counterstaining with Hoechst 33258. IGL: internal granular layer; ML: molecular layer; Nd: cerebellar nodule; PNL: pre-neoplastic lesion; Tm: tumor tissue. Scale bar: 50μm (representative for all panels).

The analysis of much younger mice (6 weeks after birth) showed that *Atoh1* is expressed at similar levels in PNLs of *Ptch1* ^Δ/+^ and *Ptch1* ^Δ/+^ *Pn-1* ^Δ/+^ mice (Fig 5A). Likewise, MAP2, which normally marks differentiating neurons, is also expressed to a similar extent in PNLs of both genotypes (Fig 5B). In contrast, only few scattered SOX9-positive cells are present in PNLs of *Ptch1* ^Δ/+^ mice (Fig 5C), while widespread SOX9 expression is apparent in PNLs of *Ptch1* ^Δ/+^ *Pn-1* ^Δ/+^ mice (Fig 5C). This early increase in SOX9 is paralleled by overexpression of GFAP (Fig 5D), while GABARα6 is not detected in PNLs (S4 Fig). This analysis shows that the GNP marker *Atoh1* remains in PNLs of *Ptch1* ^Δ/+^ *Pn-1* ^Δ/+^ mice, whereas the expression of non-GNP genes such as SOX9 and GFAP is already up-regulated at these early stages. In addition to these medulloblastoma markers, we also assessed the expression of the matrix metalloprotease MMP9 (Fig 6), which inactivates PN-1 and has been implicated in growth and malignant progression of different types of tumors including medulloblastomas (see Discussion). MMP9 proteins are detected in PNLs of both genotypes (Fig 6A) and persist in medulloblastomas, while expression appears much reduced in cerebellar nodules (Fig 6B).

### Reduced Hedgehog signal transduction and lowered cell proliferation in *Ptch1* ^Δ/+^ *Pn-1* ^Δ/+^ PNLs

**Fig 7:**
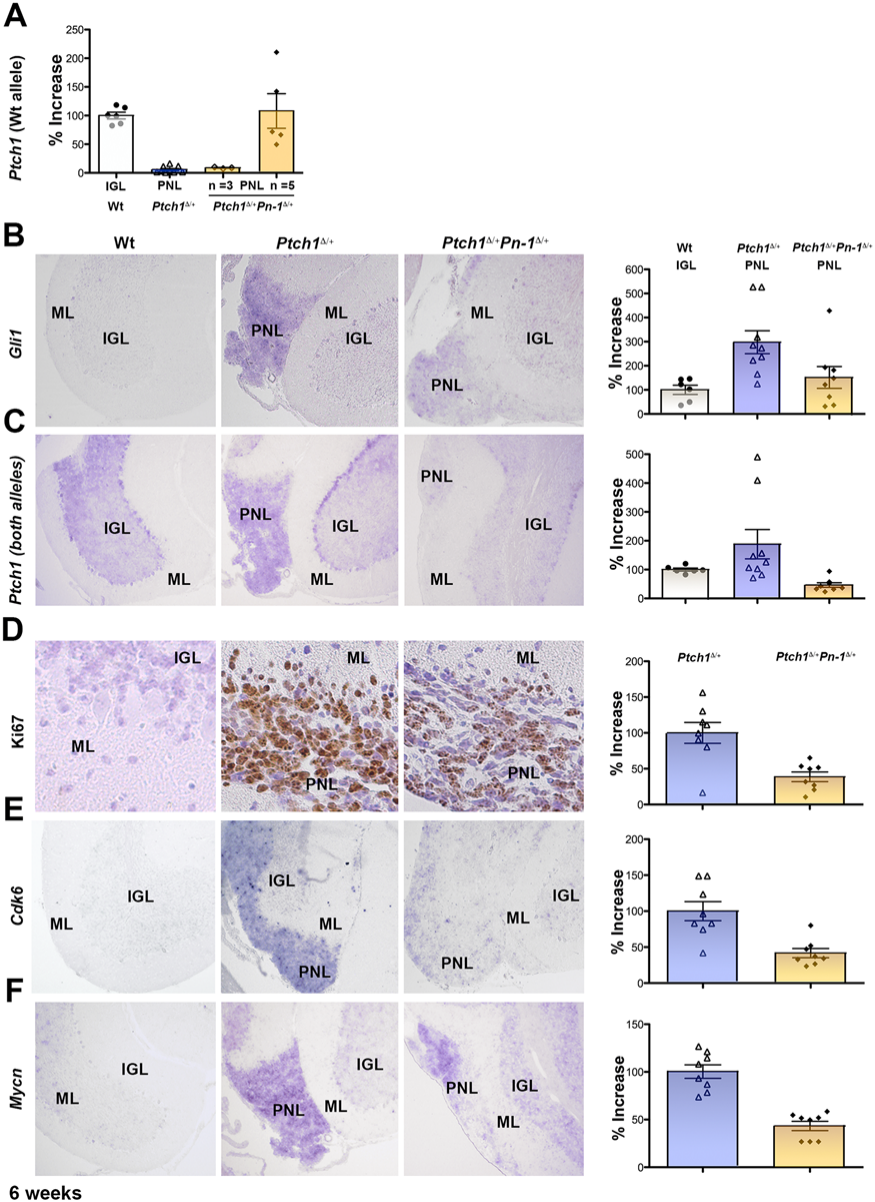
Genetic reduction of Serpine2/*Pn-1* interferes with transcriptional silencing of the wild-type *Ptch1* allele in *Ptch1* ^Δ/+^ mice resulting in reduced cell proliferation. (A) Quantitative analysis of transcripts from the wild-type *Ptch1* allele. Expression of the wild-type *Ptch1* allele is lost from all *Ptch1* ^Δ/+^ PNLs by six weeks after birth (n = 8). In contrast, the wild-type *Ptch1* allele remains expressed in 5 of 8 *Ptch1* ^Δ/+^ *Pn-1* ^Δ/+^ samples (p≤0.001). (B, C) Comparative analysis of the cerebella of wild-type (Wt), *Ptch1* ^Δ/+^ and *Ptch1* ^Δ/+^ *Pn-1* ^Δ/+^ mice at 6 weeks by RNA *in situ* hybridization and qPCR analysis (p≤0.001). As *Gli1* and *Ptch1* are direct transcriptional targets of Hedgehog signaling, their expression serves to sense signal transduction. Note that the RNA *in situ* probe detects *Ptch1* transcripts from both the mutant and wild-type *Ptch1* allele. (D) Most *Ptch1* ^Δ/+^ PNL cells are Ki67-positive (brown staining), i.e. are actively proliferating. Fewer Ki67-positive cells are detected in *Ptch1* ^Δ/+^ *Pn-1* ^Δ/+^ PNLs. (E) *Cdk6* expression is up-regulated in PNLs of *Ptch1* ^Δ/+^ mice, while it is reduced in PNLs of *Ptch1* ^Δ/+^ *Pn-1* ^Δ/+^ mice. (F) *Mycn* is overexpressed in PNLs of *Ptch1* ^Δ/+^ mice, while its expression is significantly lower in PNLs of *Ptch1* ^Δ/+^ *Pn-1* ^Δ/+^ mice. qPCR analysis shows that *Mycn*, *Cdk6* and *Ki-67* are not expressed by the IGL of wild-type mice at 6 weeks of age. Therefore, the relative expression of these three genes in PNLs of *Ptch1* ^Δ/+^ mice is set to 100% to allow comparison with the expression in *Ptch1* ^Δ/+^ *Pn-1* ^Δ/+^ PNLs (p≤0.001 for all results). IGL: internal granular layer; ML: molecular layer; PNL: pre-neoplastic lesion. Scale bar: 100μm (representative for all low magnifications shown).

Transcriptional silencing of the remaining wild-type *Ptch1* allele is a hallmark of malignant progression in *Ptch1* ^Δ/+^ mice [19,25]. Therefore, its expression was assessed using primers that only detect wild-type *Ptch1* transcripts [13,19]. In agreement with previous analyses, no expression of the wild-type *Ptch1* allele is detected in *Ptch1* ^Δ/+^ PNLs (Fig 7A, n = 8/8) [19,25]. In contrast, wild-type *Ptch1* transcripts remain in ~60% of all *Ptch1* ^Δ/+^ *Pn-1* ^Δ/+^ PNLs (Fig 7A, n = 5/8), indicating that the wild-type allele has escaped inactivation. As *Ptch1* and *Gli1* are direct transcriptional targets of HH signal transduction [54], their expression serves as transcriptional sensors of HH pathway activity [55]. Indeed, *Gli1* transcription is ~3-fold increased in *Ptch1* ^Δ/+^ PNLs compared to wild-type IGLs (Fig 7B, n = 9). In *Ptch1* ^Δ/+^ *Pn-1* ^Δ/+^ PNLs, *Gli1* expression is significantly lower than in *Ptch1* ^Δ/+^ PNLs and rather comparable to wild-type IGLs (Fig 7B, n = 7/8). A probe that detects the *Ptch1* transcripts produced by both the wild-type and mutant allele in *Ptch1* ^Δ/+^ mice reveals that *Ptch1* remains expressed in PNLs (Fig 7C) in spite of transcriptional silencing of the wild-type *Ptch1* allele (Fig 7A). Therefore, these *Ptch1* transcripts likely arise as a consequence of continued expression of the mutant *Ptch1* ^Δ^ allele (Fig 7C) [13]. In contrast, genetic reduction of *Pn-1* results in the wild-type *Ptch1* allele escaping transcriptional silencing (Fig 7A). Therefore, functional *Ptch1* transcripts remain expressed in *Ptch1* ^Δ/+^ *Pn-1* ^Δ/+^ PNLs, which results in overall lower Ptch1 levels as observed in *Ptch1* ^Δ/+^ PNLs (Fig 7C). This apparently counterintuitive result is explained by the fact that PTCH1 is a negative regulator of HH signal transduction, which in absence of ligand results in reduced *Gli1* and *Ptch1* expression as is observed (Fig 7B and 7C). Taken together, this analysis indicates that heterozygosity for *Serpine2/Pn-1* lowers the aberrantly high HH pathway activity in PNLs of *Ptch1* ^Δ/+^ mice.

During normal cerebellar development, GNPs cease to proliferate as confirmed by the absence of Ki67-positive cells in the IGL and molecular layer six weeks after birth (left panel, Fig 7D). In contrast, most cells in PNLs of *Ptch1* ^Δ/+^ mice continue to proliferate (Fig 7D, n = 8), while Ki67-positive cells were reduced by more than 50% in *Ptch1* ^Δ/+^ *Pn-1* ^Δ/+^ PNLs (Fig 7D, n = 8). In contrast, no significant increase in apoptosis was detected in PNLs and cerebellar nodules of *Ptch1* ^Δ/+^ *Pn-1* ^Δ/+^ mice, which excludes altered survival of PNL cells as underlying cause (data not shown). The significance of the reduction in proliferation of PNL cells is corroborated by molecular analysis of *Cdk6* in both genotypes (Fig 7E). *Cdk6* is a direct transcriptional target of the Hedgehog pathway, which regulates the G1-S transition of the cell cycle [55,56] and its aberrant expression in human medulloblastomas has been correlated with poor prognosis [57,58]. As expected, *Cdk6* transcripts are no longer detected in wild-type cerebella 6 weeks after birth, while high levels persist in *Ptch1* ^Δ/+^ PNLs (Fig 7E). In contrast, *Cdk6* transcript levels are reduced by ~60% in *Ptch1* ^Δ/+^ *Pn-1* ^Δ/+^ PNLs (Fig 7E, n = 8). These alterations are paralleled by significant reduction of the aberrantly high *Mycn* expression (Fig 7F), which promotes malignant progression of medulloblastomas (see Introduction). Taken together, the genetic reduction of *Pn-1* lowers the expression of genes that promote proliferation and malignant progression of medulloblastomas in *Ptch1* ^Δ/+^ mice. This analysis provides a plausible molecular explanation for the ~60% reduction in malignant medulloblastomas (Fig 3) and the rather benign nature of remaining cerebellar nodules observed in ~20% of symptom-free *Ptch1* ^Δ/+^ *Pn-1* ^Δ/+^ mice at 10–11 months of age (Fig 4 and 6B).

## Discussion

The genetic and molecular analysis analyses of mouse medulloblastomas in *Ptch1* ^Δ/+^ and *Ptch1* ^Δ/+^ *Pn-1* ^Δ/+^ mice reveals that aberrant expression of *Serpine2/Pn-1* promotes proliferation and facilitates progression of PNLs to medulloblastomas. *SERPINE2/PN-1* is expressed in most human medulloblastomas, with levels being highest in the two subgroups with altered WNT and SHH pathway activities. In addition, high levels of expression were detected in various gliomas, which agrees the normal expression of Serpine2/Pn-1 in glial cells [38]. We show that siRNA-mediated lowering of *SERPINE2/PN-1* in human medulloblastoma DAOY cells or genetically in *Ptch1* ^Δ/+^ *Pn-1* ^Δ/+^ mice reduces cell proliferation and results in the majority, but not all PNLs remaining as small cerebellar nodules rather than progression to malignant medulloblastomas. Therefore, the present analysis reveals an aberrant role for Serpine2/PN-1 in tumor growth and malignant progression, which likely complements its previously established functions in tumor invasion and metastasis [31–33,35,37]. In particular, heterozygosity for a *Pn-1* loss-of-function allele in *Ptch1* ^Δ/+^ mice lowers the aberrant Hedgehog pathway activity during medulloblastoma development. Normally Hedgehog signal transduction is tightly, partly by a transcriptional inhibitory feedback loop: the expression of the *Ptch1* receptor is up-regulated in response to SHH signaling, which results in progressive inhibition of signal transduction in situations where the ligand is limiting [59]. In PNLs of *Ptch1* ^Δ/+^ mice, this inhibitory *Ptch1* receptor feedback loop is disrupted as a consequence of silencing of the remaining wild-type *Ptch1* allele (this study). As the wild-type allele escapes transcriptional silencing in a significant fraction of *Ptch1* ^Δ/+^ *Pn-1* ^Δ/+^ PNLs, this inhibitory feedback loop remains at least partially functional, which provides a straightforward explanation for the observed lowering in Hedgehog pathway activity. Furthermore, *Atoh1* is lost from the cerebellar nodules in aged *Ptch1* ^Δ/+^ *Pn-1* ^Δ/+^ mice, which indicates that they have lost immature GNP characteristics [24], although the lack of GABARα6 expression indicates that they do not differentiate into mature cerebellar granule neurons [53]. Accordingly, the neuronal marker SYP [60] is lost from the cerebellar nodules of aged *Ptch1* ^Δ/+^ *Pn-1* ^Δ/+^ mice, while its expression is increased in end-stage *Ptch1* ^Δ/+^ medulloblastomas. Our molecular analysis of these nodules indicates that reduction of *Serpine2/Pn-1* interferes primarily with tumor cell proliferation and malignant progression, but does not promote differentiation into specific neuronal or glial cell-types. Therefore, the cerebellar nodules in aged *Ptch1* ^Δ/+^ *Pn-1* ^Δ/+^ mice seem to correspond to growth-arrested remnants of the former PNLs. As neither heterozygosity nor complete inactivation of *Serpine2/Pn-1* totally suppresses medulloblastoma development in *Ptch1* ^Δ/+^ mice, aberrant Serpine2/PN-1 appears to promote cell proliferation and malignant progression rather than being essential for medulloblastoma development. Indeed, correlation of *SERPINE2/PN-1* expression levels with survival across the entire human medulloblastoma cohort revealed only a slight difference between low and high expressors; with high *SERPINE2/PN-1* expressors surviving slightly better (p = 0.023). Furthermore, retrospective analysis of *SERPINE2/PN-1* transcript levels in patients with SHH subgroup medulloblastomas does not reveal any significant prognostic value with respect to long-term survival.

At first sight, the role of Serpine2/PN-1 appears at odds with its established role in inhibiting SHH signaling and GNP proliferation during cerebellar development [38] and its recently demonstrated functions in reducing proliferation of metastatic prostate cancer cells [36]. Molecular analysis showed that these inhibitory effects depend on SHH ligand-mediated signal transduction as LRP-1 mediated uptake of Serpine2/PN-1 results in down-regulation of *Shh* expression, which lowers Hedgehog signal transduction and cell proliferation. However, human SHH subtype and mouse *Ptch1* ^Δ/+^ medulloblastomas arise as a consequence of SHH ligand-independent activation of signal transduction due to mutations in and/or inactivation of *Ptch1* and downstream effectors (see above). Therefore, the overexpression of Serpine2/PN-1 promotes cell proliferation and malignant progression of medulloblastomas independent of its inhibitory effect on SHH ligand-mediated signal transduction. While identification of the underlying molecular mechanism requires further analysis, we noted that the expression of MMP9, which normally cleaves PN-1 and remodels the ECM is markedly reduced in cerebellar nodules of *Ptch1* ^Δ/+^ *Pn-1* ^Δ/+^ mice. This could be a consequence of disrupting PN-1 mediated up-regulation *Mmp9* expression [33]. The general role of matrix-metalloproteases in promoting tumor progression is well established [61]. In particular, the proteolytic activity of MMP9 promotes loss of tissue polarity and induces proliferation and tumor growth of human breast cancer cells in a mouse xenograft model [62]. Recently, it has been shown that MMP9 is part of a regulatory feedback loop that functions in irradiation-induced angiogenesis in medulloblastoma cells [63]. As inhibition of MMP9 blocked the aberrant cell proliferation and re-established tissue polarity [62], it is possible that the observed down-regulation of MMP9 is not only a consequence but contributes to the significant reduction in malignant progression of PNLs to medulloblastomas in *Ptch1* ^Δ/+^ *Pn-1* ^Δ/+^ mice (this study). Taken together, these studies point to the existence of two distinct mechanisms by which SerpinE2/PN-1 modulates growth and progression of tumors arising as a consequence of aberrant Hedgehog pathway activity. Malignant progression of SHH ligand-dependent tumors such as prostate adenocarcinomas would be inhibited by high Serpine2/PN-1 [36], while the malignancy of SHH ligand-independent tumors such as medulloblastomas (this study) and gastric cancer [64] would be aggravated. Identification of the distinct molecular interactions underlying these two mechanisms will not only help to clarify the rather complex functions of Serpine2/PN-1 in modulating signaling and tumor progression, but also reveal to what extent this extra-cellular protein may be suited-or not- for therapeutic intervention. Assessing the potential suitability of Serpine2/PN-1 as a therapeutic target is important, as inhibiting ligand-independent activation of the HH pathway by small molecule SMO antagonists results in drug resistance. This is due to selection of tumor cells with a corresponding mutation in *SMO*, which results in recurrence of medulloblastomas and metastasis [65]. Therefore, it might be beneficial to combine antagonism of SMO with Serpine2/PN-1 inhibitors to help preventing relapse due to single drug resistance.

## Supporting Information

S1 Fig *SERPINE2/PN-1* expression is upregulated in various brain tumors. (A) Expression levels of *SERPINE2/PN-1*in the four medulloblastoma subgroups (red) in comparison to other brain tumors (blue) and normal controls (normal cerebellum: grey; normal CNS and non-CNS tissues: green). The expression data for medulloblastomas, other brain tumors and normal tissues were compiled from multiple gene expression profiling studies [5,39–47] (Kool et al. unpublished data). Each dot represents one sample. All data were generated by Affymetrix Human U133plus2.0 arrays and were MAS5.0 normalized. Grey bars represent mean values. (B) Positive correlation of *SERPINE2/PN-1* with *PTCH1* and Sox9 expression in SHH subtype medulloblastomas. The R2 microarray analysis and visualization platform (http://r2.amc.nl) was used to order the samples according to their *SERPINE2/PN-1* expression levels and for statistical verification. Red dots represent the samples ordered by their *SERPINE2/PN-1* expression levels, while the blue dots indicate the corresponding *PTCH1* and *SOX9* expression levels, respectively. For the correlation of *SERPINE2/PN-1* with *PTCH1* the statistical significance is p = 4.0e-07, r = 0.556 (indicating moderate positive correlation); for *SERPINE2/PN-1* with *SOX9* the values are p = 1.7e-12, r = 0.715 (indicating strong positive correlation).
(TIF)

S2 Fig Expression of SerpinE2/PN-1 and additional marker proteins in human medulloblastoma biopsies. (A) Immuno-detection of the KI67 antigen reveals proliferating tumor cells in the human medulloblastoma biopsy shown in Fig 1B. Analysis of Synaptophysin (SYP), MAP2, SOX9 and GFAP on sections of the same biopsy. (B) Haematoxylin-eosin (HE) staining and analysis of the PN-1, KI67, SYP and MAP2 protein distributions in a second representative human medulloblastoma biopsy. Scale bar: 150μm.
(TIF)

S3 Fig *Pn-1* transcript and PN-1 protein distribution in PNLs of *Ptch1* ^Δ/+^ mice. (A) *Pn-1* transcripts are detected by RNA *in situ* hybridization (purple staining) in PNLs of *Ptch1* ^Δ/+^ mice at 3 weeks postnatally already. Frame indicates the area magnified in the right panel. (B) PN-1 protein distribution in PNLs of *Ptch1* ^Δ/+^ and *Ptch1* ^Δ/+^ *Pn-1* ^Δ/+^ mice detected by immunohistochemistry at 6 weeks (brown staining). IGL: internal granular layer; ML: molecular layer; PCL: Purkinje cell layer; PNL: pre-neoplastic lesion. Scale bars: 250μm (left panel in A); 50μm (right panel in A and both panels in B).
(TIF)

S4 Fig The expression the GABA receptor α6-subunit (GABARα6), a marker for mature granule neurons is not up-regulated in cerebellar nodules. (A) Distribution of the GABARα6 protein (brown stained immunocomplexes) in the cerebellum of *Ptch1* ^Δ/+^ mice at 6 weeks (upper panel) and 6 months (lower panel) of age. (B) Distribution of the GABARα6 protein in the cerebellum of *Ptch1* ^Δ/+^ *Pn-1* ^Δ/+^ mice at 6 weeks (upper panel) and 6 months (lower panel) of age. All mature granule neurons of the IGL express the GABARα6 protein, while PNLs, medulloblastomas and cerebellar nodules are negative. Nuclei appear white fluorescent due to counterstaining with DAPI. IGL: internal granular layer; ML: molecular layer; Nd: cerebellar nodule; PCL: Purkinje cell layer; PNL: pre-neoplastic lesion, Tm: tumor tissue. Scale bar: 100μm (representative for all sections shown).
(TIF)

S1 Table Excel table of 410 genes. These are genes, whose expression correlates significantly (p<0.001) with the one of SERPINE2/PN-1 in human medulloblastomas of the SHH subtype (n = 119).
(XLSX)

S2 Table Primary and secondary antibodies. (DOCX)

S3 Table Primers for qPCR analysis. (DOCX)
