# Supplementary figures and images for: Serpine2/PN-1 Is Required for Proliferative Expansion of Pre-Neoplastic Lesions and Malignant Progression to Medulloblastoma
Source: PLoS One. 2015 Apr 22;10(4):e0124870. doi: 10.1371/journal.pone.0124870 (PMC4406471; doi:10.1371/journal.pone.0124870)

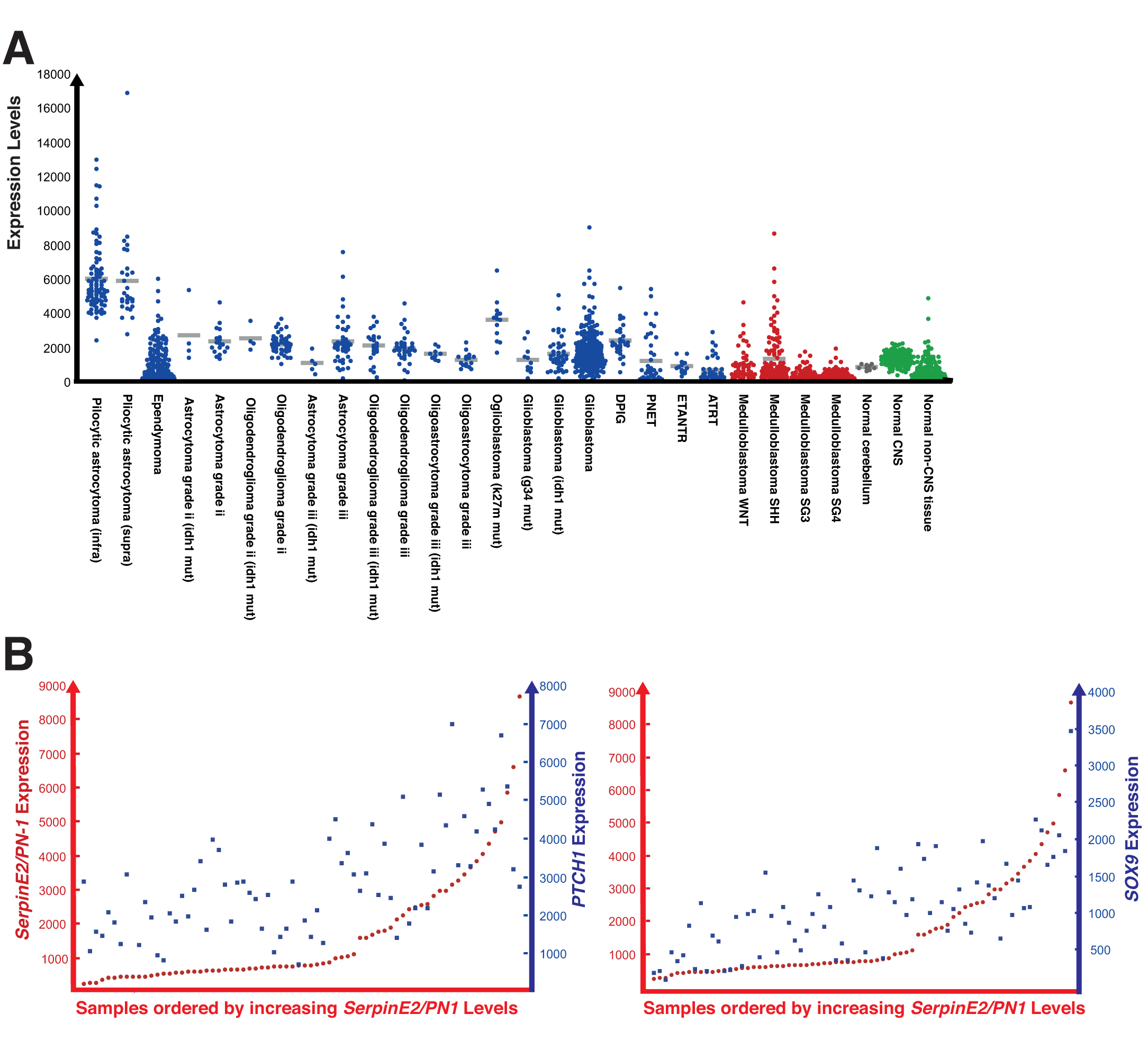

Supplement: S1 Fig — (A) Expression levels of SERPINE2/PN-1in the four medulloblastoma subgroups (red) in comparison to other brain tumors (blue) and normal controls (normal cerebellum: grey; normal CNS and non-CNS tissues: green). The expression data for medulloblastomas, other brain tumors and normal tissues were compiled from multiple gene expression profiling studies [5,39–47] (Kool et al. unpublished data). Each dot represents one sample. All data were generated by Affymetrix Human U133plus2.0 arrays and were MAS5.0 normalized. Grey bars represent mean values. (B) Positive correlation of SERPINE2/PN-1 with PTCH1 and Sox9 expression in SHH subtype medulloblastomas. The R2 microarray analysis and visualization platform (http://r2.amc.nl) was used to order the samples according to their SERPINE2/PN-1 expression levels and for statistical verification. Red dots represent the samples ordered by their SERPINE2/PN-1 expression levels, while the blue dots indicate the corresponding PTCH1 and SOX9 expression levels, respectively. For the correlation of SERPINE2/PN-1 with PTCH1 the statistical significance is p = 4.0e-07, r = 0.556 (indicating moderate positive correlation); for SERPINE2/PN-1 with SOX9 the values are p = 1.7e-12, r = 0.715 (indicating strong positive correlation). (TIF) [file pone.0124870.s001.tif]

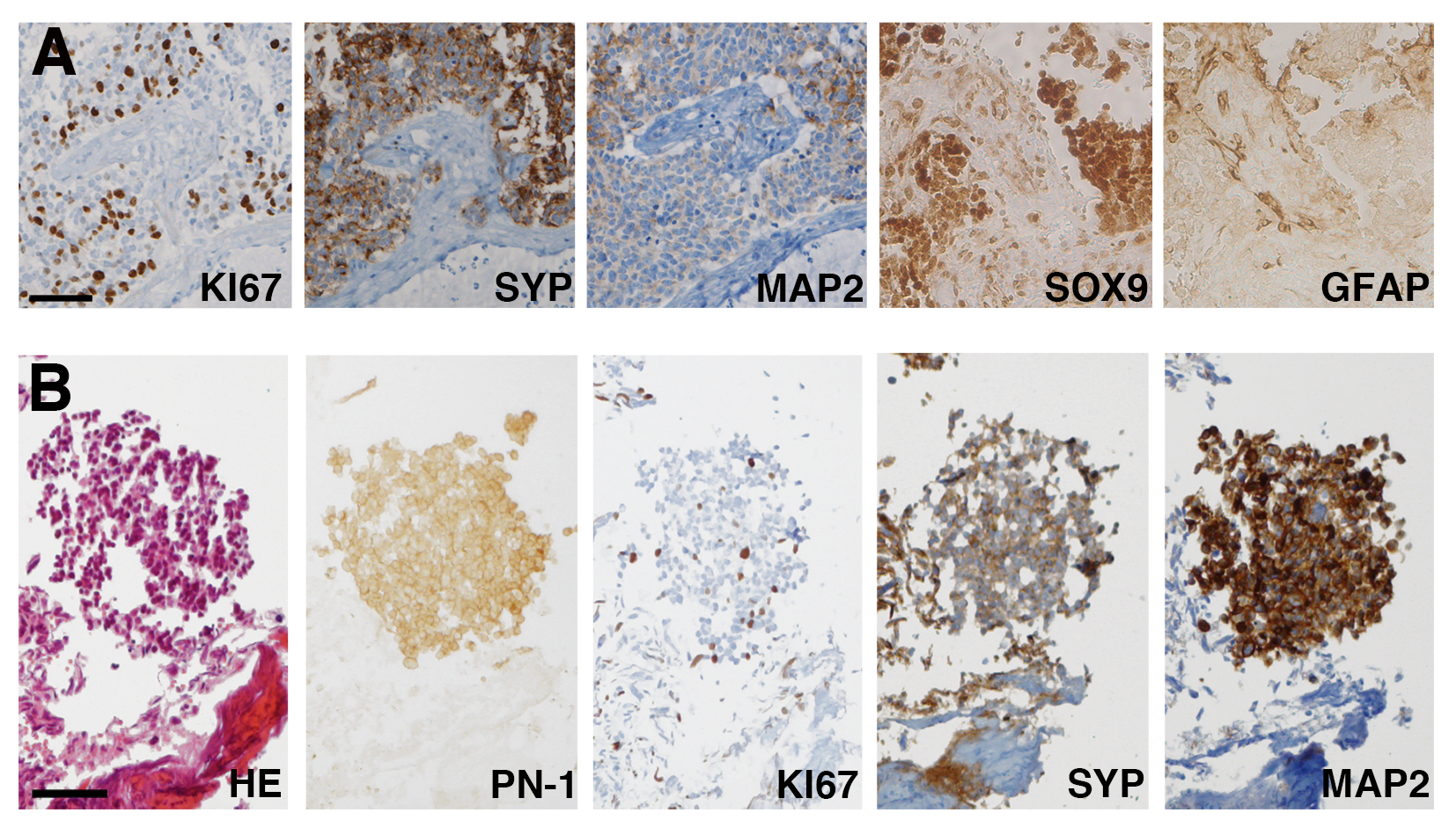

Supplement: S2 Fig — (A) Immuno-detection of the KI67 antigen reveals proliferating tumor cells in the human medulloblastoma biopsy shown in Fig 1B. Analysis of Synaptophysin (SYP), MAP2, SOX9 and GFAP on sections of the same biopsy. (B) Haematoxylin-eosin (HE) staining and analysis of the PN-1, KI67, SYP and MAP2 protein distributions in a second representative human medulloblastoma biopsy. Scale bar: 150μm. (TIF) [file pone.0124870.s002.tif]

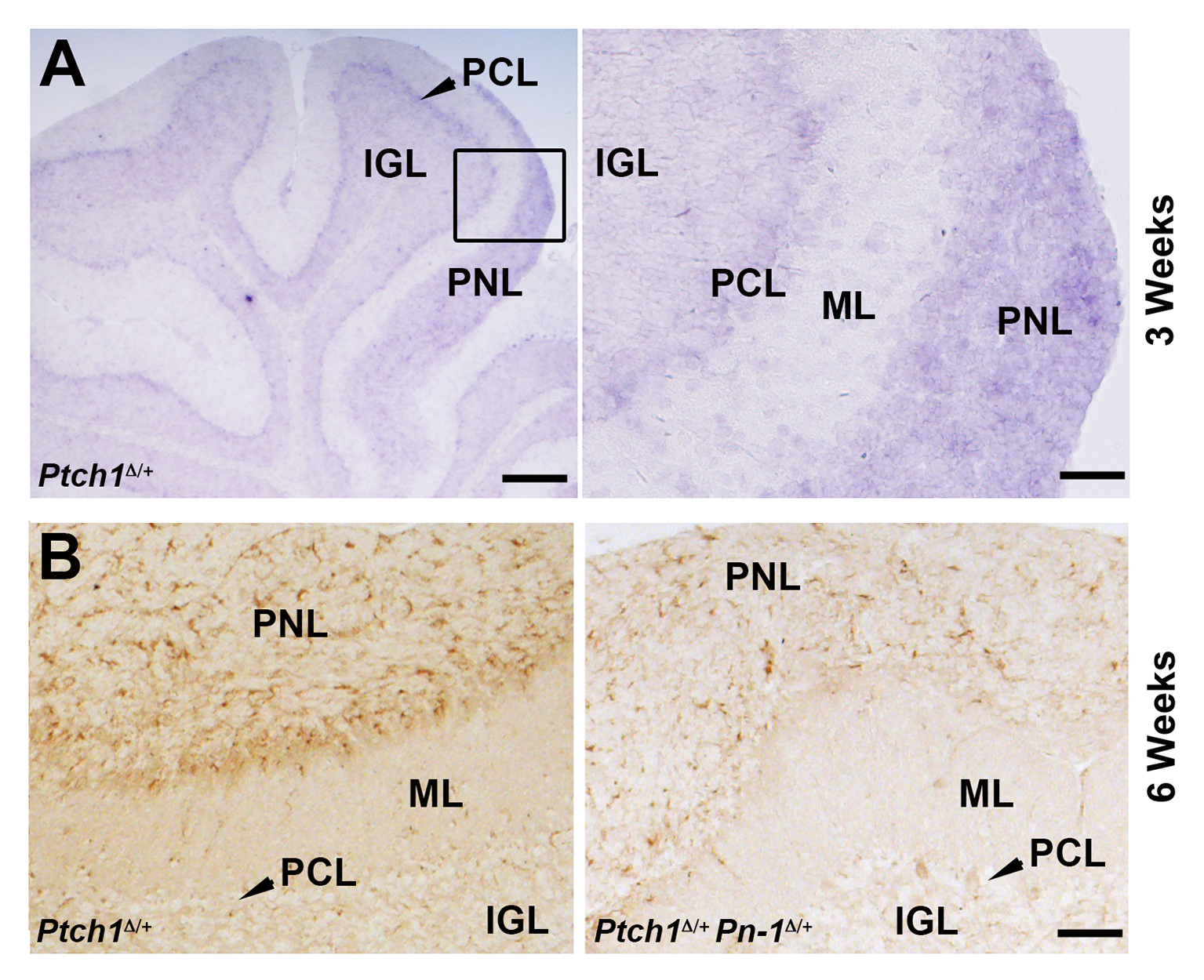

Supplement: S3 Fig — (A) Pn-1 transcripts are detected by RNA in situ hybridization (purple staining) in PNLs of Ptch1 Δ/+ mice at 3 weeks postnatally already. Frame indicates the area magnified in the right panel. (B) PN-1 protein distribution in PNLs of Ptch1 Δ/+ and Ptch1 Δ/+ Pn-1 Δ/+ mice detected by immunohistochemistry at 6 weeks (brown staining). IGL: internal granular layer; ML: molecular layer; PCL: Purkinje cell layer; PNL: pre-neoplastic lesion. Scale bars: 250μm (left panel in A); 50μm (right panel in A and both panels in B). (TIF) [file pone.0124870.s003.tif]

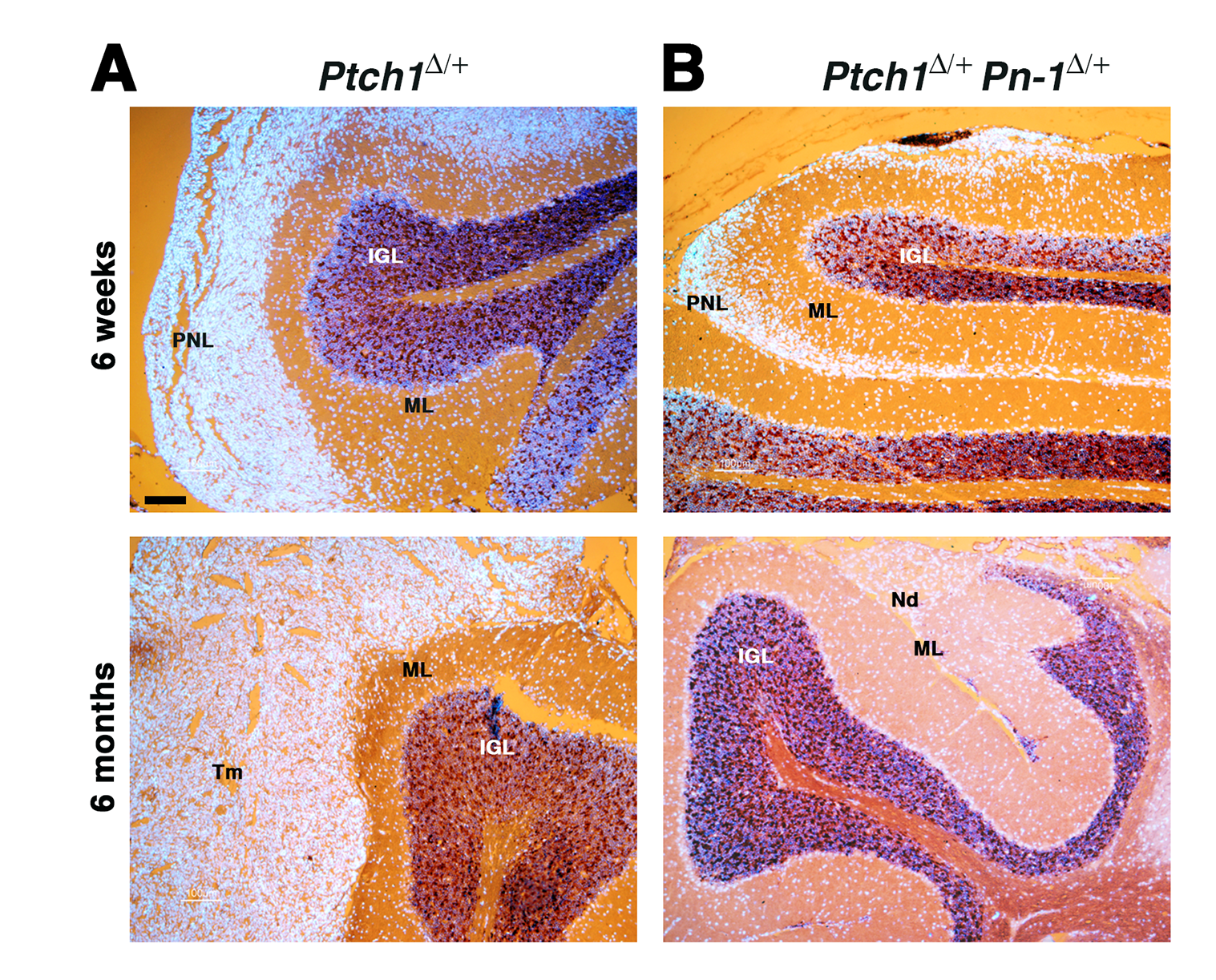

Supplement: S4 Fig — (A) Distribution of the GABARα6 protein (brown stained immunocomplexes) in the cerebellum of Ptch1 Δ/+ mice at 6 weeks (upper panel) and 6 months (lower panel) of age. (B) Distribution of the GABARα6 protein in the cerebellum of Ptch1 Δ/+ Pn-1 Δ/+ mice at 6 weeks (upper panel) and 6 months (lower panel) of age. All mature granule neurons of the IGL express the GABARα6 protein, while PNLs, medulloblastomas and cerebellar nodules are negative. Nuclei appear white fluorescent due to counterstaining with DAPI. IGL: internal granular layer; ML: molecular layer; Nd: cerebellar nodule; PCL: Purkinje cell layer; PNL: pre-neoplastic lesion, Tm: tumor tissue. Scale bar: 100μm (representative for all sections shown). (TIF) [file pone.0124870.s004.tif]
