# Supplementary material for: Serpine2/PN-1 Is Required for Proliferative Expansion of Pre-Neoplastic Lesions and Malignant Progression to Medulloblastoma
Source: PLoS One. 2015 Apr 22;10(4):e0124870. doi: 10.1371/journal.pone.0124870 (PMC4406471; doi:10.1371/journal.pone.0124870)
Supplement: S2 Table — (DOCX) [file pone.0124870.s006.docx]

**S2 Table. Primary and secondary antibodies**

| **Primary antibodies** | **Manufacturer** | **Applications** |
| --- | --- | --- |
| α-SerpinE2 (PN-1) | Proteintech | IF/IB |
| α-SOX9 | Millipore | IF/IHC/IB |
| α-Vinculin | Sigma | IB |
| α-BrdU (G3G4) | DSHB | IF |
| α-rat PN1 (clone 4B3) | ([1](#_ENREF_1)) | IHC |
| α-human PN-1 (clone 1F6) | ([2](#_ENREF_2)) | IHC |
| α-GFAP | Dako | IHC |
| α-human KI67 (clone MIB-1) | Dako | IHC |
| α-mouse Ki67 | Millipore | IHC |
| α-human MAP2 (clone HM-2) | Sigma | IHC |
| α-human SYP (clone 27G12) | Leica | IHC |
| α-mouse GABA A Receptor alpha 6 | Millipore | IF |
| α-MMP9 (AB19016, polyclonal) | Millipore | IF |
|  |  |  |
| **Secondary antibodies** |  |  |
| Alexa Fluor 488 donkey α-mouse IgG | Invitrogen | IF |
| Alexa Fluor 594 donkey α-rabbit IgG | Invitrogen | IF |
| Biotin-SP-conjugated donkey α-rat IgG | Dianova | IF |
| CyTM3-conjugated α-rat IgG | Dianova | IF |
| Goat α-mouse, HRP-coupled | Sigma | IB |
| Goat α-rabbit, HRP-coupled | Invitrogen | IB |
| Goat biotinylated α-mouse | Vector Laboratories | IHC |
| Goat biotinylated α-rabbit | Vector Laboratories | IHC |

IB: immunoblotting, IF: immunofluorescence, IHC: immunohistochemistry
