# Supplementary material for: Serpine2/PN-1 Is Required for Proliferative Expansion of Pre-Neoplastic Lesions and Malignant Progression to Medulloblastoma
Source: PLoS One. 2015 Apr 22;10(4):e0124870. doi: 10.1371/journal.pone.0124870 (PMC4406471; doi:10.1371/journal.pone.0124870)
Supplement: S3 Table — (DOCX) [file pone.0124870.s007.docx]

**S3 Table.** **Primers for qPCR analysis**

| **Mouse Gene** | **Orientation** | **Oligo Sequence** | **No. of Cycles set to 100% Expression** |
| --- | --- | --- | --- |
| ***Cdk6*** | **forward**  **reverse** | **5’ AGG TAA GGG CCA TCT GAA AAC T 3’**  **5’ GGC GTA CCC ACA GAA ACC ATA 3’** | **29** |
| ***Gli1*** | **forward**  **reverse** | **5’ CAA GTG CAC GTT TGA AG 3’**  **5’ CAA CCT TCT TGC TCA CAC ATG TAA G 3’** | **29** |
| ***Hprt1*** | **forward**  **reverse** | **5’ ACT TTG CTT TCC CTG GT 3’**  **5’ AGC TTG CTG GTG AAA AGG ACC 3’** | **29** |
| ***Ki67*** | **forward**  **reverse** | **5’ GCT CGC CTT GAT GGT TCC T 3’**  **5’ ATC ATT GAC CGC TCC TTT AGG T 3’** | **29** |
| ***Mycn*** | **forward**  **reverse** | **5’ TGC TGC TGA TGG ATG GGA AC 3’**  **5’ AAC AAG GCG GTA ACC ACT TTC AC 3’** | **27** |
| ***Pn-1*** | **forward**  **reverse** | **5’ GAA GCA GCT CTC CAC GGT 3’**  **5’ AGA CAA TAG CCT TGT TGA TC 3’** | **29** |
| ***Ptch1 (Exon 2-3)*** | **forward**  **reverse** | **5’ CCT CTT CTC CTA TCT TCT GAC GGG 3’**  **5’ GGC AAG TTT TTG GTT GTG GGT C 3’** | **32** |
| ***Ptch1 (Exon 7-9)*** | **forward**  **reverse** | **5’ GCA CCT TTT GAG TGG AGT TTG G 3’**  **5’ CAT TGG CAG GAG GAG TTG ATT G 3’** | **30** |
